# Supplementary material for: Coordinated Regulation of Central Carbon Metabolism in Pyroligneous Acid-Treated Tomato Plants under Aluminum Stress
Source: Metabolites. 2023 Jun 20;13(6):770. doi: 10.3390/metabo13060770 (PMC10303050; doi:10.3390/metabo13060770)
Supplement: Supplementary file 1 [file metabolites-13-00770-s001.zip › Figures S1-S2.pdf]

Figure S1. Chromatograms for all identified metabolites of CCM routes. The sections (1-3) show detection of CCM metabolites. The compound names and the ion transition of each compound are shown on the extracted ion chromatograms (XICs)

1. TCA cycle

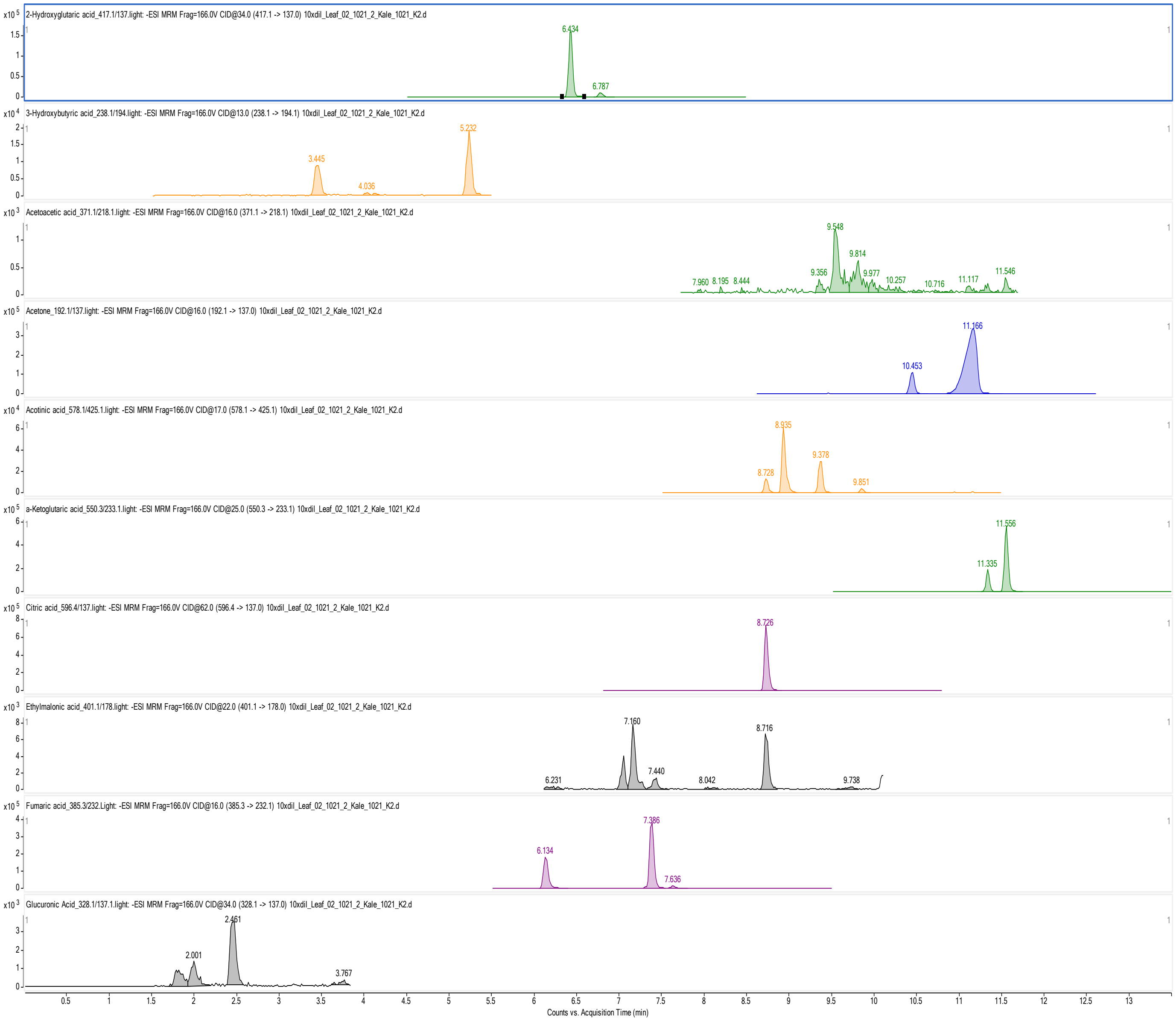

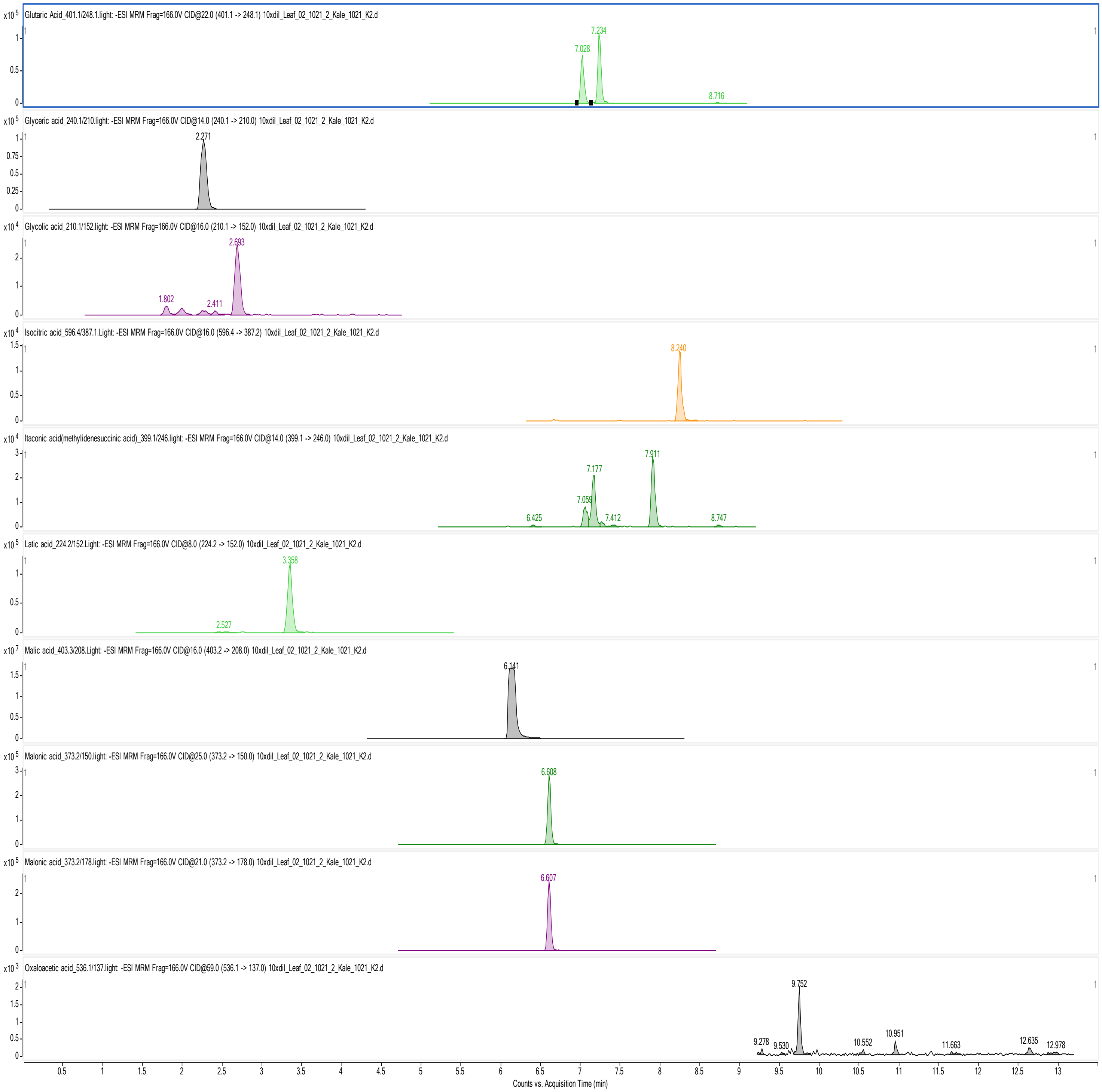

Glutaric Acid_401.1/248.1.light: -ESI MRM Frag=166.0V CID@22.0 (401.1 -> 248.1) 10xdil_Leaf_02_1021_2_Kale_1021_K2.d
7.234
7.028
8.716
Glyceric acid_240.1/210.light: -ESI MRM Frag=166.0V CID@14.0 (240.1 -> 210.0) 10xdil_Leaf_02_1021_2_Kale_1021_K2.d
2.271
Glycolic acid_210.1/152.light: -ESI MRM Frag=166.0V CID@16.0 (210.1 -> 152.0) 10xdil_Leaf_02_1021_2_Kale_1021_K2.d
2.693
1.802
2.411
Isocitric acid_596.4/387.1.Light: -ESI MRM Frag=166.0V CID@16.0 (596.4 -> 387.2) 10xdil_Leaf_02_1021_2_Kale_1021_K2.d
8.240
Itaconic acid(methylidenesuccinic acid)_399.1/246.light: -ESI MRM Frag=166.0V CID@14.0 (399.1 -> 246.0) 10xdil_Leaf_02_1021_2_Kale_1021_K2.d
7.911
7.177
7.059
6.425
7.412
8.747
Latic acid_224.2/152.Light: -ESI MRM Frag=166.0V CID@8.0 (224.2 -> 152.0) 10xdil_Leaf_02_1021_2_Kale_1021_K2.d
3.358
2.527
Malic acid_403.3/208.Light: -ESI MRM Frag=166.0V CID@16.0 (403.2 -> 208.0) 10xdil_Leaf_02_1021_2_Kale_1021_K2.d
6.141
Malonic acid_373.2/150.light: -ESI MRM Frag=166.0V CID@25.0 (373.2 -> 150.0) 10xdil_Leaf_02_1021_2_Kale_1021_K2.d
6.608
Malonic acid_373.2/178.light: -ESI MRM Frag=166.0V CID@21.0 (373.2 -> 178.0) 10xdil_Leaf_02_1021_2_Kale_1021_K2.d
6.607
Oxaloacetic acid_536.1/137.light: -ESI MRM Frag=166.0V CID@59.0 (536.1 -> 137.0) 10xdil_Leaf_02_1021_2_Kale_1021_K2.d
9.752
9.278
9.530
10.552
10.951
11.663
12.635
12.978
Counts vs. Acquisition Time (min)

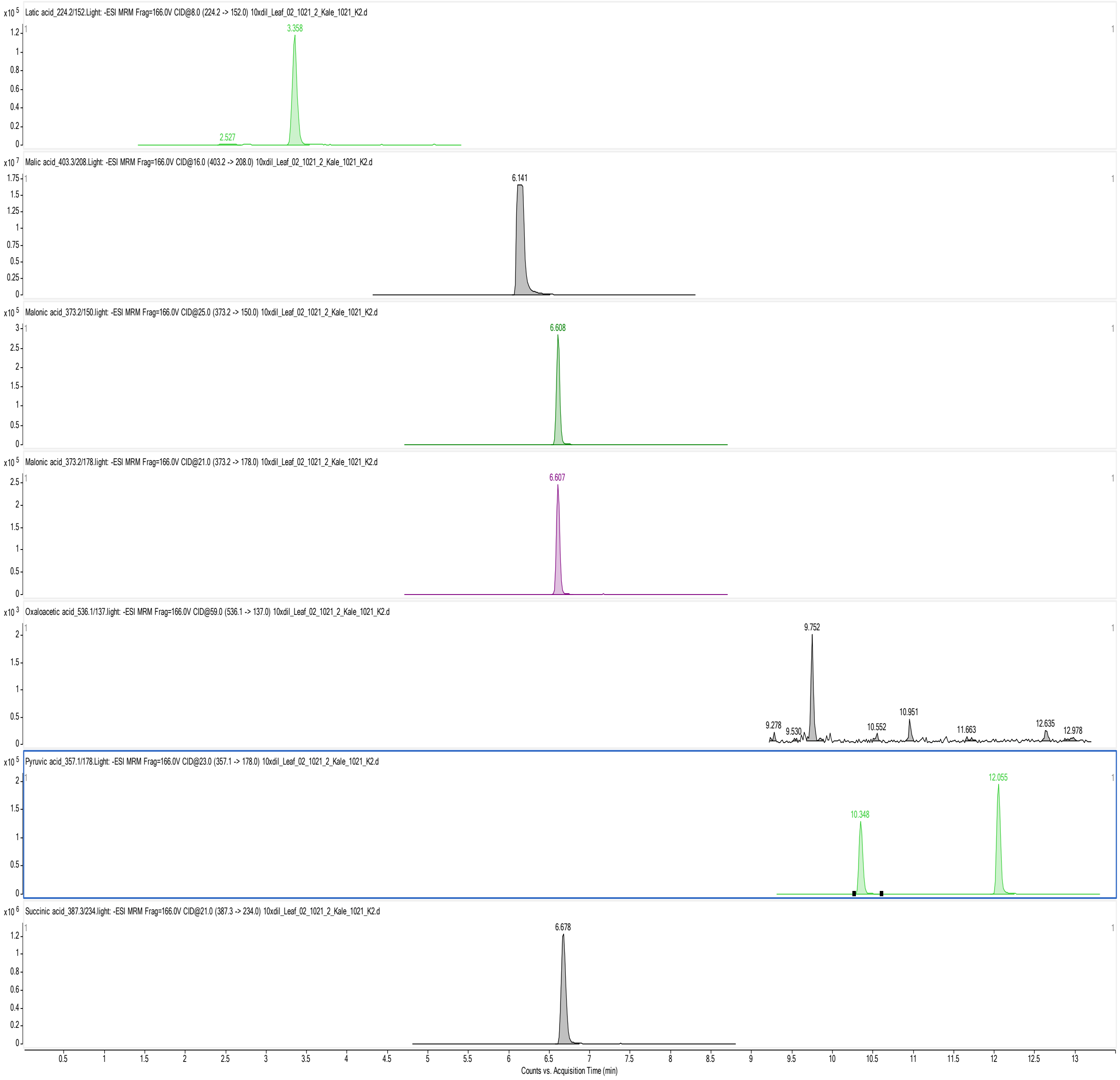
Latic acid_224.2/152.Light: -ESI MRM Frag=166.0V CID@8.0 (224.2 -> 152.0) 10xdil_Leaf_02_1021_2_Kale_1021_K2.d
Malic acid_403.3/208.Light: -ESI MRM Frag=166.0V CID@16.0 (403.2 -> 208.0) 10xdil_Leaf_02_1021_2_Kale_1021_K2.d
Malonic acid_373.2/150.light: -ESI MRM Frag=166.0V CID@25.0 (373.2 -> 150.0) 10xdil_Leaf_02_1021_2_Kale_1021_K2.d
Malonic acid_373.2/178.light: -ESI MRM Frag=166.0V CID@21.0 (373.2 -> 178.0) 10xdil_Leaf_02_1021_2_Kale_1021_K2.d
Oxaloacetic acid_536.1/137.light: -ESI MRM Frag=166.0V CID@59.0 (536.1 -> 137.0) 10xdil_Leaf_02_1021_2_Kale_1021_K2.d
Pyruvic acid_357.1/178.Light: -ESI MRM Frag=166.0V CID@23.0 (357.1 -> 178.0) 10xdil_Leaf_02_1021_2_Kale_1021_K2.d
Succinic acid_387.3/234.light: -ESI MRM Frag=166.0V CID@21.0 (387.3 -> 234.0) 10xdil_Leaf_02_1021_2_Kale_1021_K2.d
3.358
2.527
6.141
6.608
6.607
9.752
9.278
9.530
10.552
10.951
11.663
12.635
12.978
10.348
12.055
6.678
Counts vs. Acquisition Time (min)

2. Sugars and selected sugar phosphates

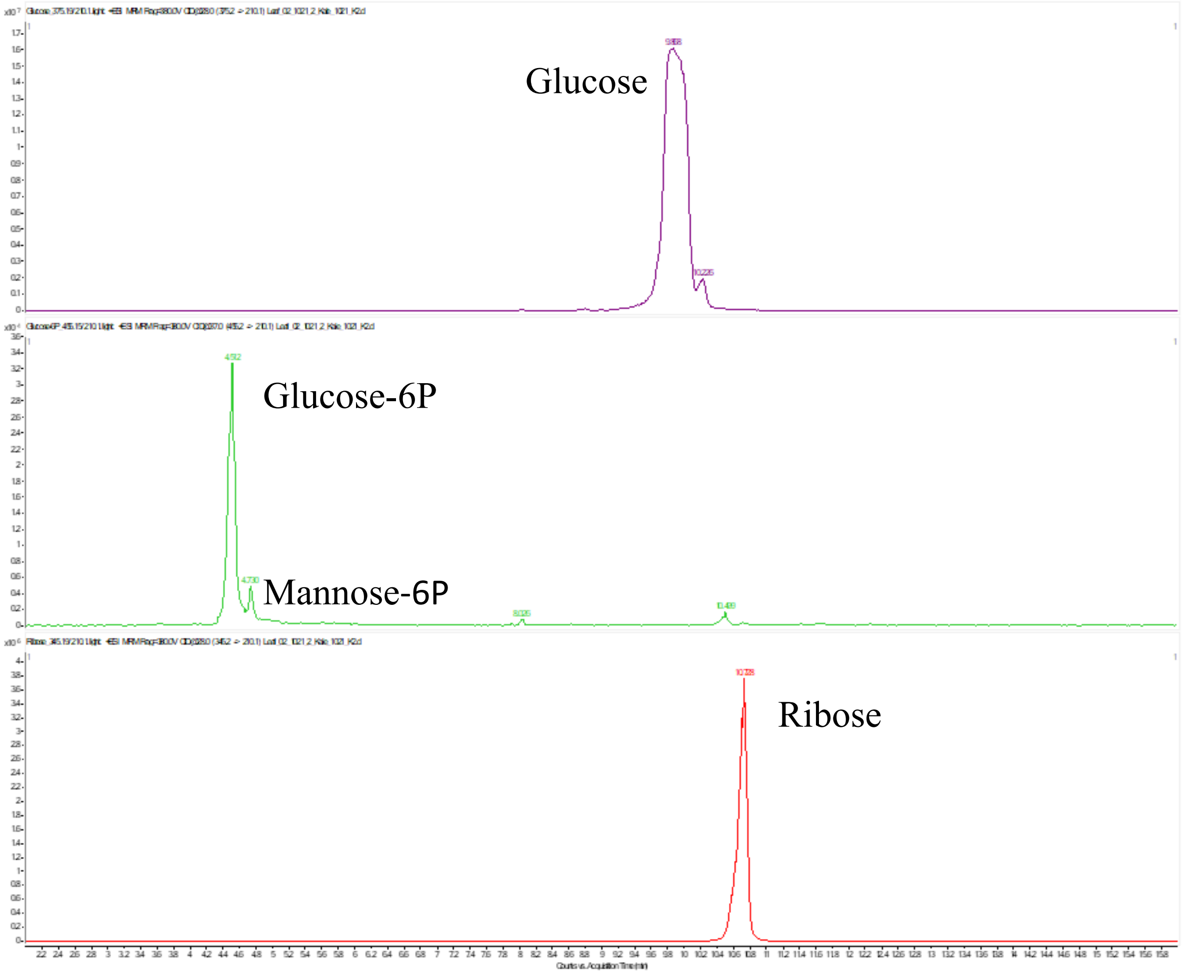

3. Phosphometabolites

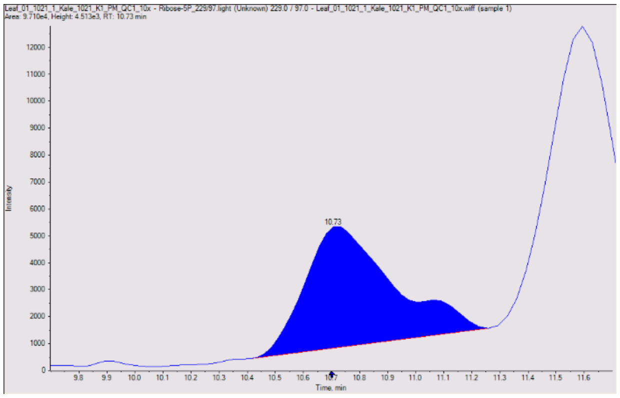

Ribose-5P

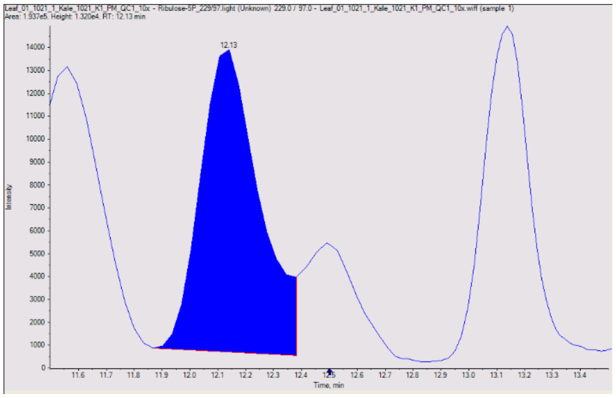

Ribulose-5P

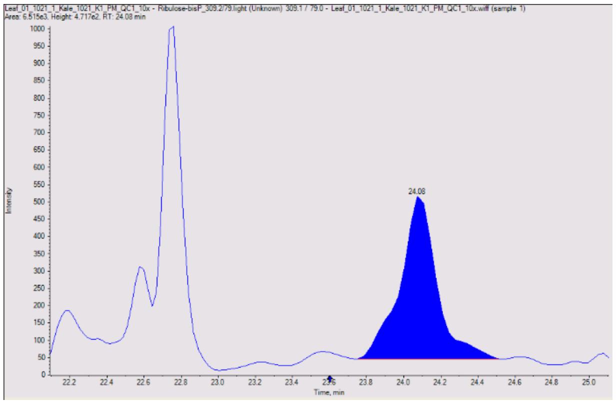

Ribulose-bisP

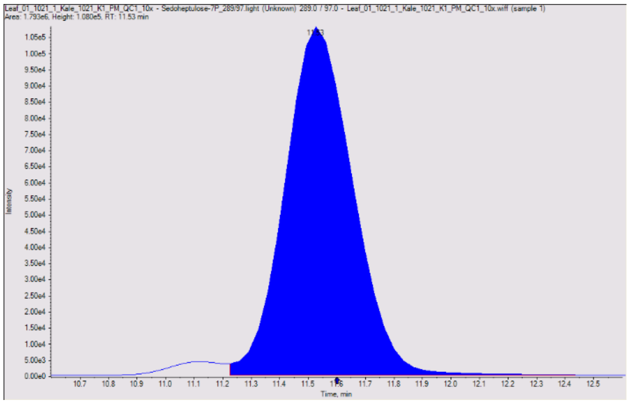

Sedoheptulose-7P

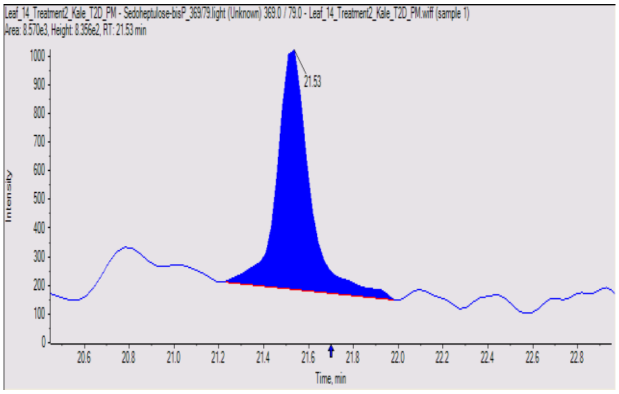

Sedoheptulose-1,7-bisP

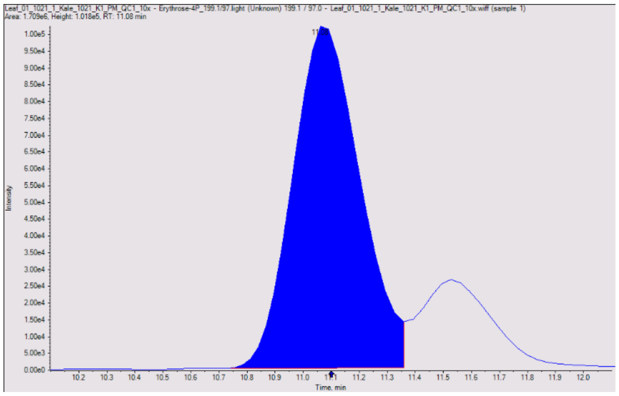

Erythrose-4P

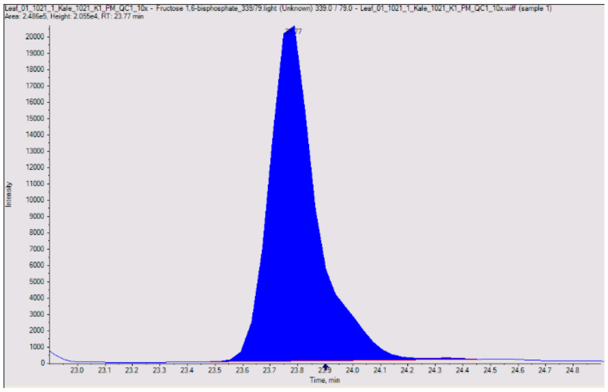

Fructose-1,6-bisP

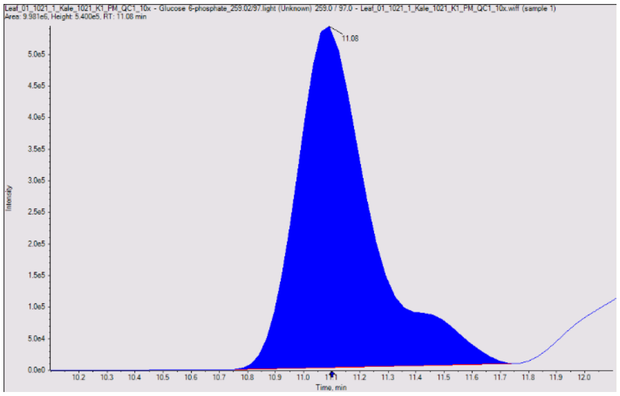

Fructose-6P

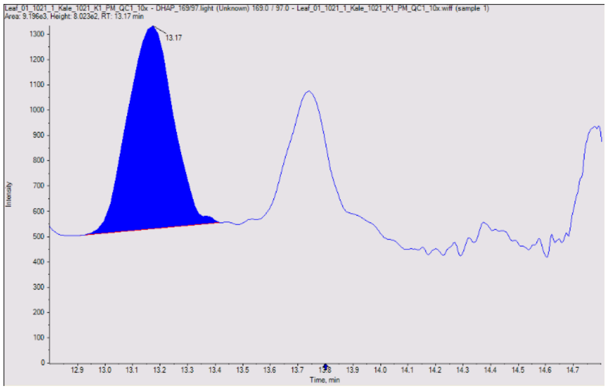

DHAP

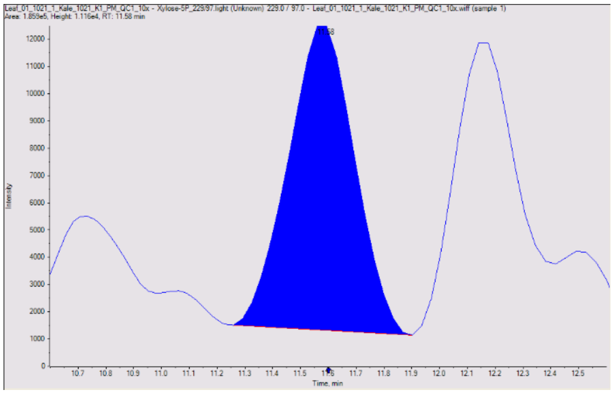

Xylose-5P

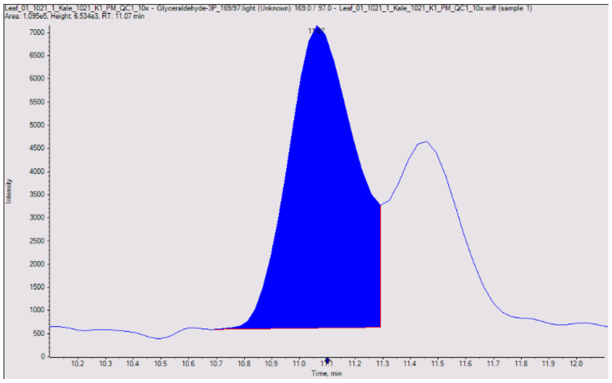

Glyceraldehyde-3P

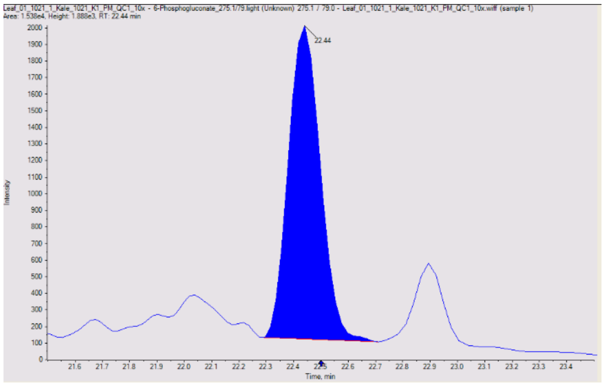

6-Phosphogluconate

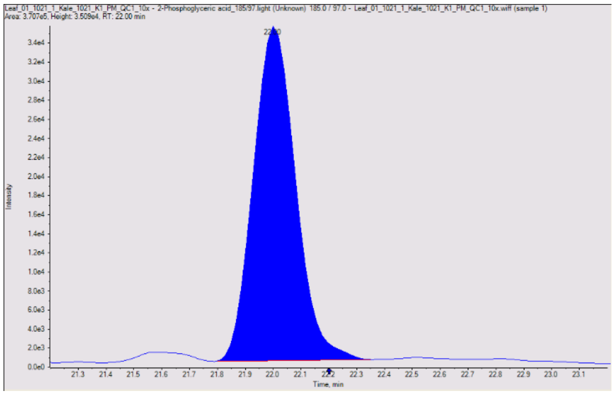

3-Phosphoglyceric acid

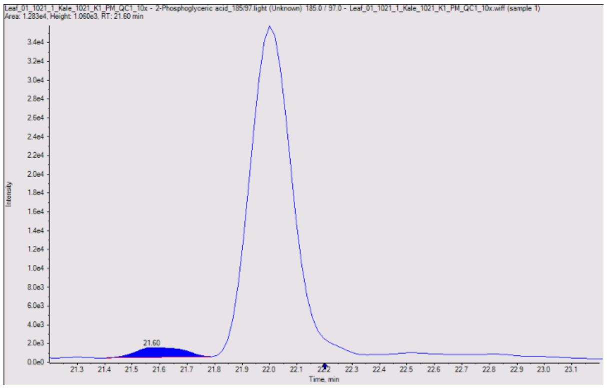

2-Phosphoglyceric acid

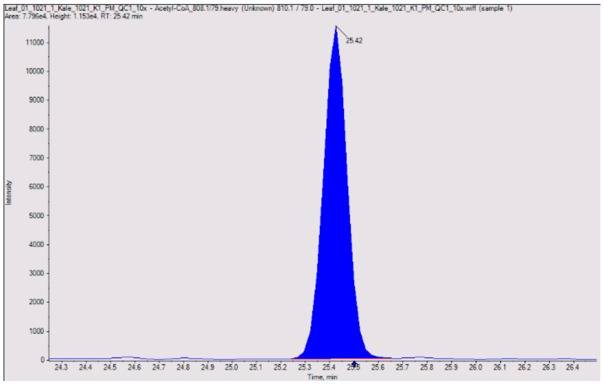

Acetyl-CoA

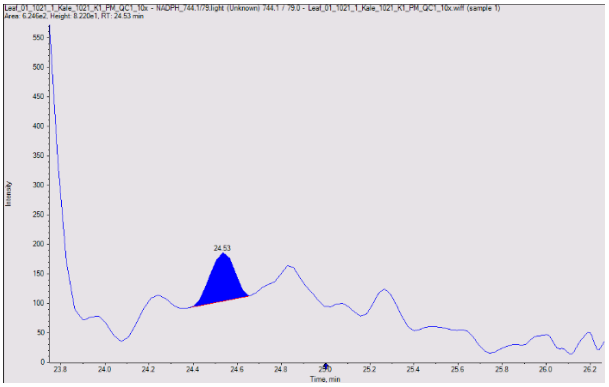

NADPH

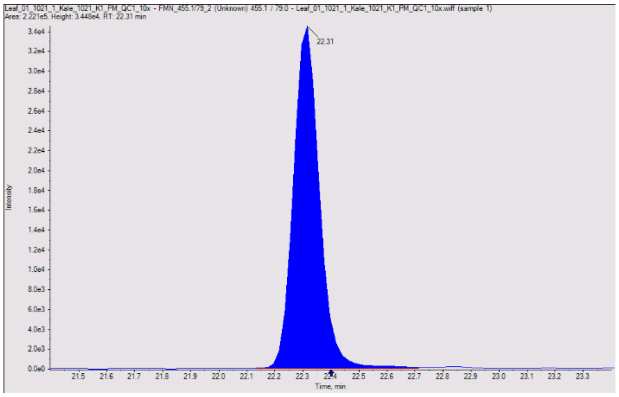

FMN

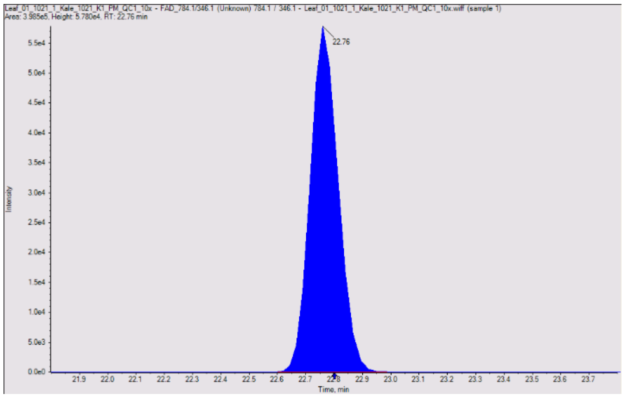

FAD

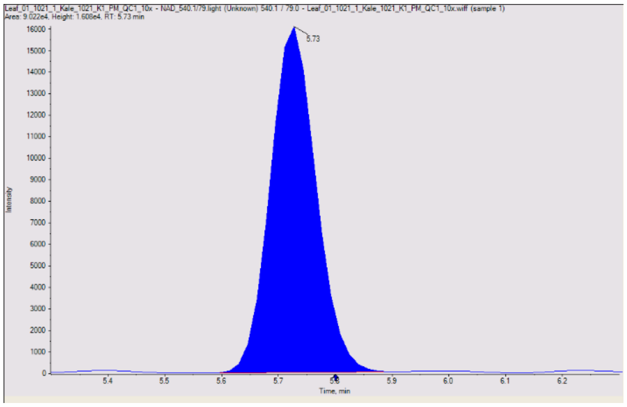

NAD

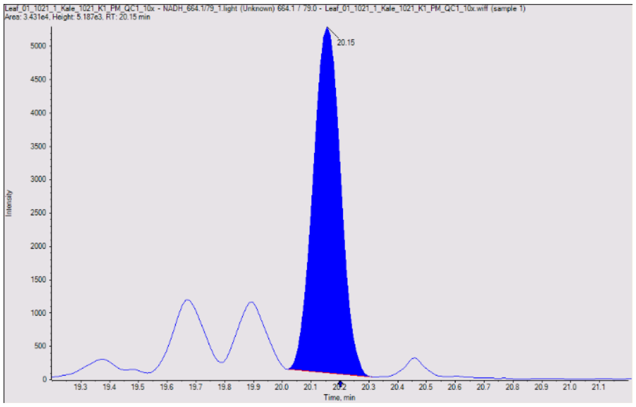

NADH

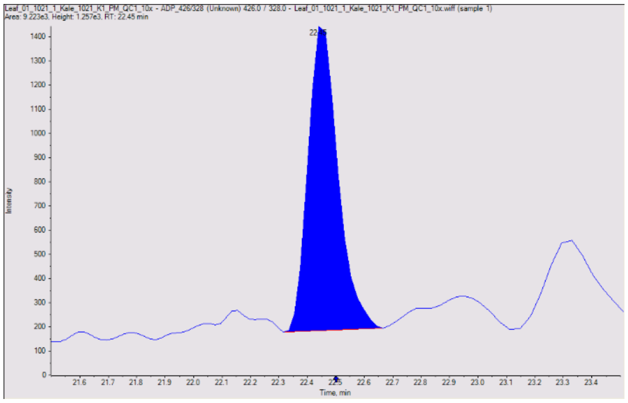

ADP

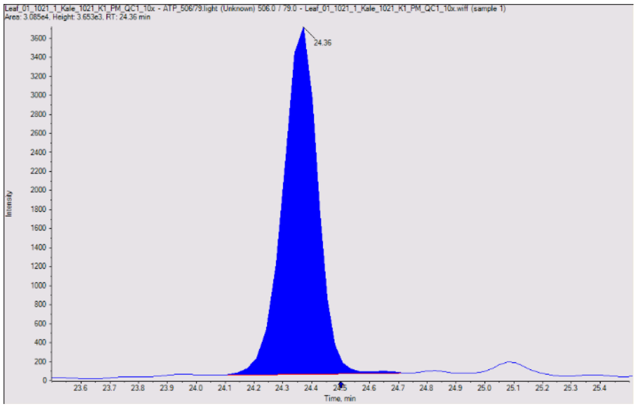

ATP

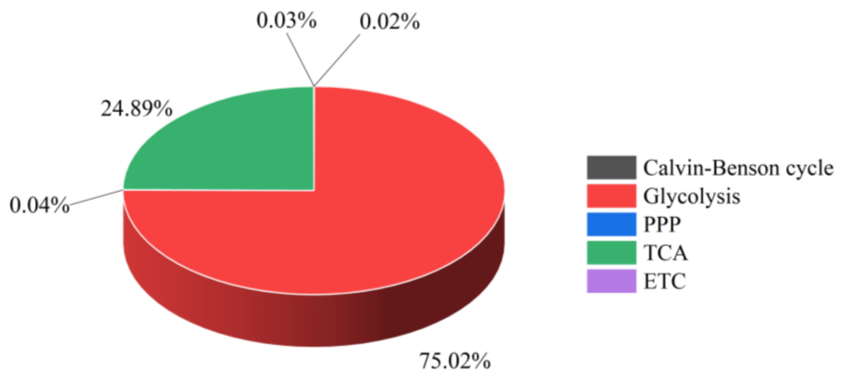

Figure S2. Classification of metabolites involved in CCM route (Calvin–Benson cycle, glycolysis, pentose phosphate pathway (PPP), tricarboxylic acid (TCA) cycle and electron transport chain (ETC)) in leaves of tomato 'Scotia' seedlings treated with pyroligneous acid (PA) under aluminum (Al) stress.
